# Postnatal changes and sexual dimorphism in collagen expression in mouse skin

**DOI:** 10.1371/journal.pone.0177534

**Published:** 2017-05-11

**Authors:** Koji Y. Arai, Takuya Hara, Toyofumi Nagatsuka, Chikako Kudo, Sho Tsuchiya, Yoshihiro Nomura, Toshio Nishiyama

**Affiliations:** Scleroprotein Research Institute, Faculty of Agriculture, Tokyo University of Agriculture and Technology, Fuchu, Tokyo, Japan; Medical University of South Carolina, UNITED STATES

## Abstract

To investigate sexual dimorphism and postnatal changes in skin collagen expression, mRNA levels of collagens and their regulatory factors in male and female skin were examined during the first 120 days of age by quantitative realtime PCR. Levels of mRNAs encoding extracellular matrices did not show any differences between male and female mice until day 15. *Col1a1* and *Col1a2* mRNAs noticeably increased at day 30 and remained at high levels until day 120 in male mice, while those in female mice remained at low levels during the period. Consistent with the mRNA expression, pepsin-soluble type I collagen contents in skin was very high in mature male as compared to female. *Col3a1* mRNA in male mice also showed significantly high level at day 120 as compared to female. On the other hand, expression of mRNAs encoding TGF-ßs and their receptors did not show apparent sexual dimorphism although small significant differences were observed at some points. Castration at 60 days of age resulted in a significant decrease in type I collagen mRNA expression within 3 days, and noticeably decreased expression of all fibril collagen mRNAs examined within 14 days, while administration of testosterone tube maintained the mRNA expression at high levels. Despite the in vivo effect of testosterone, administration of physiological concentrations of testosterone did not affect fibril collagen mRNA expression in either human or mouse skin fibroblasts in vitro, suggesting that testosterone does not directly affect collagen expression in fibroblasts. In summary, present study demonstrated dynamic postnatal changes in expression of collagens and their regulatory factors, and suggest that testosterone and its effects on collagen expression are responsible for the skin sexual dimorphism but the effects of testosterone is not due to direct action on dermal fibroblasts.

## Introduction

We empirically know sexual dimorphism of skin in mammals including humans. Male’s skin is generally thick and hard as compared to female’s skin. The features of male skin would be suitable for fighting against other males to increase the chance for mating. Many studies demonstrated differences in skin features between men and women [1]. For example, skin collagen density is low in women as compared to men at all ages examined (15–93 years old) [2]. Sex differences in skin features also demonstrated in experimental animals. Dermis of male mouse skin is significantly thicker than female’s dermis [3, 4], and male mouse skin contains high amounts of hydroxyproline [4, 5] and collagen [4] as compared to female. In addition to the sexual dimorphism, it is generally recognized that features of skin, such as thickness and elasticity, change during postnatal development [1]. Skin collagen density decreases with age [2]. As well as collagen density, human skin thickness decreases in elderly skin [2, 6]. Furthermore, a previous study using human dermal fibroblasts from various aged women revealed that production of types I and III collagens decreased with age [7].

Extracellular matrix composition is thought to be one of the important factors affecting the skin features. Collagen fibrils, which are mainly constituted of types I, III, and V collagens, are known as major components of the dermal extracellular matrix. Type I collagen is composed of two α1(I) and one α2(I) chains, and the most abundant member of the collagen family [8]. Another major skin fibrillar collagen, type III collagen is a homotrimer which is comprised of three α1(III) chains [8]. Type III collagen is colocalized with type I collagen and regulates diameter of type I collagen fibrils [9]. In human fetal skin, type III collagen comprises 30–60% of total collagen, while type III collagen in adult skin comprises 10–20% [10]. Another study demonstrated that ratio of type III to type I collagen in dermis decreased during fetal development [11]. In addition, proportion of type III collagen compared with type I collagen decreases with age in rat skin [12]. These data suggest that the changes in the type III/type I ratio affect developmental changes in skin features. On the other hand, type V collagen is a quantitatively minor component of predominant type I collagen fibrils. The most abundant and widely distributed isoform is composed of two α1(V) and one α2(V) chains, which forms heterotypic fibrils [8]. A study of *Col5α1*-deficient mice [13] revealed a critical role of type V collagen in early fibril initiation. The mice showed vulnerable skin and abnormal hair follicle formation [13]. The study demonstrated that type V collagen is important in the determination of fibril structure and matrix organization [13]. With respect to type IV collagen, it is known as a main basement membrane composition. The most abundant and widely distributed isoform is composed of two α1(IV) and one α2(IV) chains [8]. A previous immunohistochemical study demonstrated that Type IV collagen content in human skin decreased with aging [14].

In addition to biosynthesis, deposition of collagen in tissues is regulated by post-translational degradation and modification. In humans, two tissue collagenases, matrix metalloproteinase-1 (MMP1) and MMP13, have been identified, whereas three tissue collagenases, MMP1A, MMP1B, and MMP13 have been reported in mice [15]. On the other hand, post-translational formation of collagen cross-linking stabilizes collagen fibers [16]. Activity of collagen cross-linking enzymes such as lysil oxidase and lysine hydroxylases is thought to affect collagen deposition in tissues.

Expression of extracellular matrix is well known to be regulated by transforming growth factor-ßs (TGF-ßs), which belong to the TGF-ß superfamily [15]. Three isoforms, TGF-ß1-3, are present in mammalian tissues. TGF-ß stimulates extracellular matrix expression in many tissues including skin [16]. Previous studies indicate that TGF-ß-induced fibrosis is mediated by connective tissue growth factor (CTGF), a cysteine rich protein belonging to the connective tissue growth factor/cysteine-rich 61/nephroblastoma overexpressed (CCN) family [17]. Activins, other members of the TGF-ß superfamily, are also involved in extracellular matrix expression in several tissues [18], and is up-regulated during skin wound healing [19–21]. Activities of activins are negatively regulated by follistatin and follistatin-like 3, which are activin binding proteins belonging to the follistatin family [22, 23]. Based on the previous studies, we hypothesized that members of the TGF- ß superfamily and their related factors are possible to be responsible for the sex differences in skin features.

There are apparent sexual differences and developmental changes in the feature of the skin. However, precise sexual differences and developmental changes in extracellular matrix and growth factor gene expression in the skin are not fully understood. Therefore, in the present study, we examined expression of mRNAs encoding extracellular matrices, extracellular matrix-related enzymes, members of the TGF-ß superfamily and their related proteins in male and female skin during mouse development. We also examined involvement of testosterone in the regulation of these factors both in vivo and in vitro.

## Materials and methods

### Animals

Mice of the C57BL/6J strain were purchased from CLEA Japan Inc. (Tokyo, Japan) and maintained at our laboratory. The mice were kept under controlled temperature (25±2°C) and lighting (12-h light/dark cycles) conditions, and weaned around 30 days of age. Food and water were available ad libitum. Male and female mice at 0, 5, 10, 15, 30, 60 and 120 days of age were used. Mice at 10 days of age or older were killed by cervical dislocation under isoflurane anesthesia and hair was removed by hair clipper. Mice at 0 and 5 days of age were anesthetized with ice and killed by decapitation. Dorsal skin was collected and immediately immersed in liquid nitrogen and stored at -80°C until isolation of total RNA or analysis for collagen contents. Total RNA was isolated with Trizol regent (Invitrogen, Carlsbad, CA, USA) according to the manufacture’s protocol and quantified by spectrophotometric measurement at 260nm. A part of the skin was subjected to histological observation after staining with hematoxylin and eosin. All experimental procedures using laboratory animals were approved by the Animal Experiment Committee of Tokyo University of Agriculture and Technology (approval number 26–101).

### Monolayer cell culture

Newborn human foreskin fibroblasts were purchased from KURABO Industries Ltd. (Osaka, Japan). Mouse skin fibroblasts were obtained from newborn C57BL/6J mouse skin. The newborn mice were anesthetized with ice, sterilized with 70% ethanol, and skin was collected after decapitation. The skin was washed twice with phosphate-buffered saline (PBS), and floated on Dulbecco’s Modified Eagle’s Medium (DMEM)-low glucose (1000 mg/L, Sigma-Aldrich, St. Louis, Mo, USA) containing 0.05% collagenase (type IV, Sigma-Aldrich) and 1% PSN Antibiotic Mixture (5 mg penicillin/ml, 5 mg streptomycin/ml, 10 mg neomycin/ml, Invitrogen) overnight at 4°C. On the next day, epidermal layer was separated from dermal layer. The dermal layer was dispersed by pipetting, and the dispersed tissue was collected by centrifugation at 500xg for 5 min. The collected pellet was resuspended in DMEM containing 10% fetal bovine serum (FBS) and 1% PSN Antibiotic Mixture, and dermal cells were plated on a 60-mm tissue culture plates (two mice per plate). When the cells became confluent, cells were collected and stored in liquid nitrogen until use. The mouse dermal fibroblasts within three passages were used for this study. Human and mouse fibroblasts were grown in DMEM containing 10% FBS and 1% PSN Antibiotic Mixture at 37°C in a humidified atmosphere of 95% air and 5% CO_2_. Fibroblasts were cultured in 6-well culture plates in the growing medium. When the cells became confluent, medium was removed and replaced with DMEM containing 1% dextran-charcoal-treated FBS after washing with PBS twice. After 24 h, medium was removed again and replaced with DMEM containing 1% dextran-charcoal-treated FBS, and the cells were cultured in the presence or absence of testosterone (1 or 5 ng/ml, Wako Pure Chemical Inc., Tokyo, Japan). Twenty-four hours, 72h, and 1 week after the incubation, total RNA was isolated with Trizol reagent.

### Skin equivalent model

Skin equivalents were prepared as reported previously [21]. Newborn human foreskin keratinocytes and fibroblasts were purchased from KURABO. The medium for skin-equivalent culture was composed of a 1:1 mixture of DMEM and EGF-free HuMedia-KG2 supplemented with 5% dextran-charcoal-treated FBS, 1.8 mM Ca^2+^ (final concentration), and 250 μM 2-O-a-D-glucopyranosyl-L-ascorbic acid (Wako). The medium was changed three times a week. The skin-equivalents were cultured in the presence of a proteinase inhibitor aprotinin (10 μM, Sigma-Aldrich) and an MMP inhibitor N-Hydroxy-2(R)-[[(4-methoxyphenyl)sulfonyl](3-picolyl)amino]-3-methylbutanamide hydrochloride, CGS27023A (10 μM, kindly provided by Dr. Amano, Shiseido Ltd.) from day 5 through day 14 (day 0 = the day after inoculation of keratinocytes). The skin equivalents were cultured either in the presence or absence of 5 ng/ml testosterone from day 0. At day 14 of the culture, the epidermal and dermal layers were separated with forceps, and the dermal layers were quickly frozen in liquid nitrogen. The samples were stored at -80°C until isolation of total RNA.

### Sodium dodecyl sulphate-polyacrylamide gel electrophoresis (SDS-PAGE) of skin samples

Mouse skin samples were weighed (wet weight) and collected in 1.5 ml microtubes. Ice-cold 0.1 M acetic acid containing 1 mg/ml pepsin was added to the tubes (1ml/100mg wet weight tissue). The skin samples were incubated at 4°C for one week with rotation to digest non-collagenous proteins. After centrifugation at 4°C, 14000xg, supernatants were mixed with 3x SDS-PAGE loading buffer (187.5 mM Tris-Cl [pH 6.8], 6% SDS, 30% glycerol, 0.01% bromophenol blue) at a ratio of 2:1. After reduction by dithiothreitol (final concentration, 100 mM), the samples were boiled at 100°C for 3min, and loaded on a 6% sodium dodecyl sulphate-polyacrylamide gel containing 3.3M urea. Urea was added to separate α1(III) chain from α1(I) chain according to a previous paper [24]. The pepsin-treated skin sample corresponding to 3 mg of the tissue (days 0–15) or 0.35 mg of the tissue (30–120) was applied to the SDS-PAGE. After electrophoresis, gels were stained with cumassie brilliant blue 250G and intensity of bands was analyzed with an image analyzing software Image J.

### Castration

Male mice were castrated at 60 days of age under isoflurane anesthesia. After the castration, each mouse was subcutaneously implanted with either an empty silicone tube (1 cm length, 3 mm outer and 2mm inner diameters, IMAMURA Co., Ltd., Tokyo, Japan) or a silicone tube with the same size filled with testosterone crystal at ventral skin. Both sides of the tubes (1mm each side) were sealed with dental silicone resin (Exafine Injection Type, GC Dental Products Corp., Aichi, Japan). Some animals were sham-operated and subcutaneously implanted with empty tubes. Before, and at 3, 7, and 14 days after the surgery, mice were killed by cervical dislocation under isoflurane anesthesia, and dorsal skin was collected and immersed in liquid nitrogen, and stored at -80C until isolation of total RNA.

### Quantification of mRNAs by realtime PCR

Messenger RNA levels were measured by quantitative realtime PCR as described previously [21]. Upper and lower primers were selected from different exons to avoid amplification of genomic DNA. Primers used were listed in S1 Table. To calculate absolute quantity of mRNAs, standard cDNA solutions were prepared with pGEM-T easy vectors (Promega) containing appropriate cDNAs. Complementary DNAs for the standards were obtained by RT-PCR and subjected to sequence analysis. The plasmids were quantified by spectrophotometric measurement at 260 nm, and copy numbers were calculated from their base compositions. All mRNA levels were normalized with levels of mitochondrial ribosomal protein L19 (*MRPL19 or Mrpl19*) mRNA.

### Histological analysis

Skin samples at days 10, 15, 30, 60 and 120 of age were fixed in 4% paraformaldehyde-phosphate buffered saline overnight, embedded in paraffin, and subjected to hematoxylin and eosin staining. In each mouse, five points were randomly selected in the skin section, and thickness of dermal layer was measured at the selected points. The average of the thickness at the selected points was designated as the dermal thickness of each mouse.

### Statistical analysis

Values are represented as means ± SEM. To compare two groups, Student’s t-test was used. To compare the mean values of more than three groups, results were subjected to one-way analysis of variance followed by Tukey-kramer test. In some cases, logarithmic conversion was carried out before the analysis. Differences were considered significant at *P*<0.05.

## Results

### Developmental changes in dermal thickness of male and female mice

**Fig 1:**
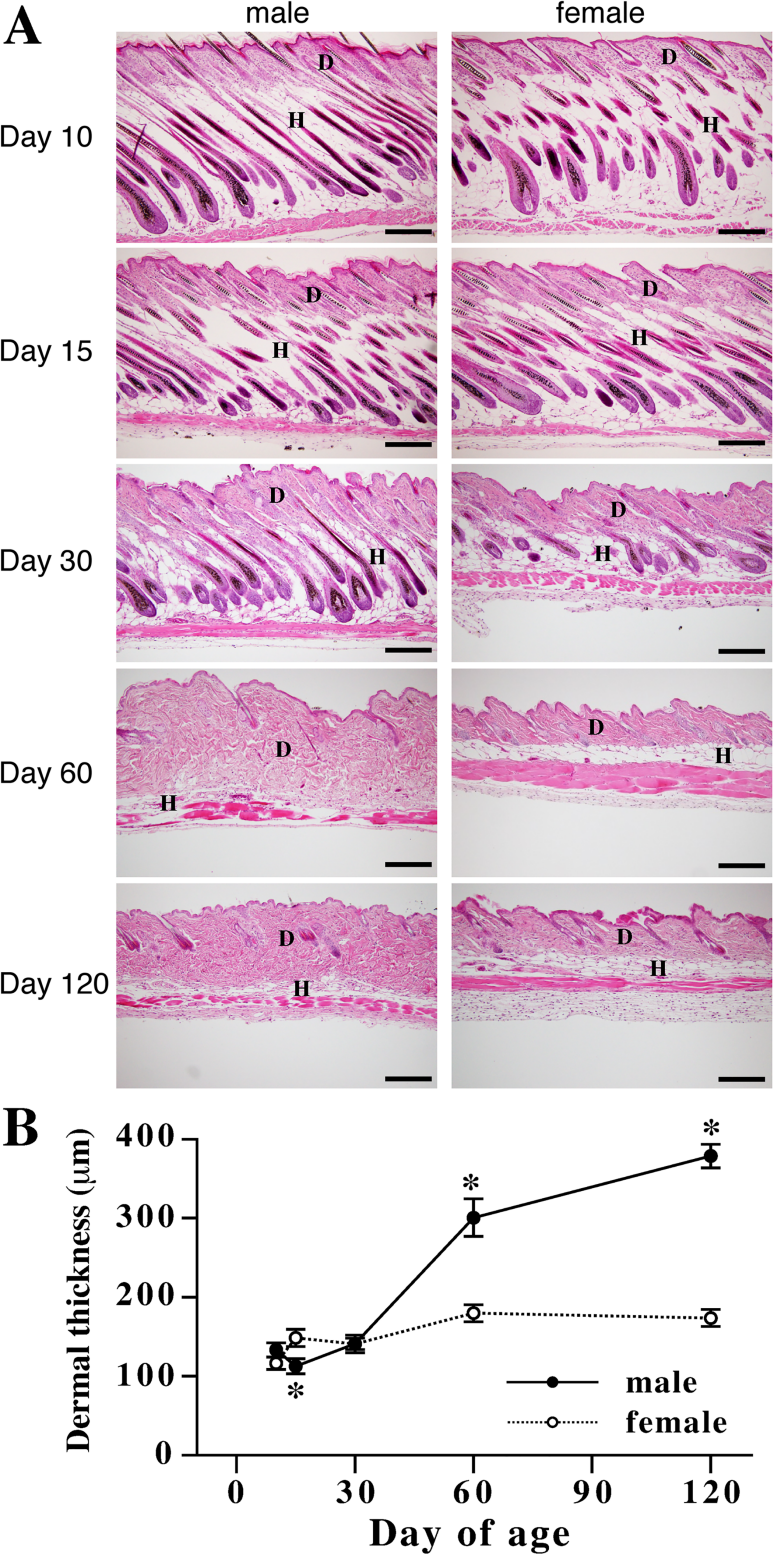
Changes in dermal thickness during mouse development. (A) Representative images of male and female skin sections stained with hematoxylin and eosin. At days 10, 15, 30, 60, and 120 of age, dorsal skin of male and female mice was collected. D indicates dermis and H indicates hypodermis. Bars indicate 200 μm. (B) Changes in dermal thickness of male (closed circles) and female (open circles) mice during development. Dermal thickness was measured at random positions of the sections. Five points were randomly selected for measuring dermal thickness in each sample, and average of the thickness of five points were designated as the dermal thickness of each sample. Values are means ± SEM for five or six animals. *, significant as compared to female animals at the same age (*P*<0.05).

Noticeable differences in dermal thickness were not observed between male and female until 30 days of age although dermis in female mice at day 15 was significantly thicker than male. In mature mice, i.e. mice at 60 and 120 days of age, the thickness of male dermis was nearly or more than twice of that of female dermis (Fig 1).

### Postnatal changes in extracellular matrix and extracellular matrix-related enzyme mRNA expression in male and female mouse skin

**Fig 2:**
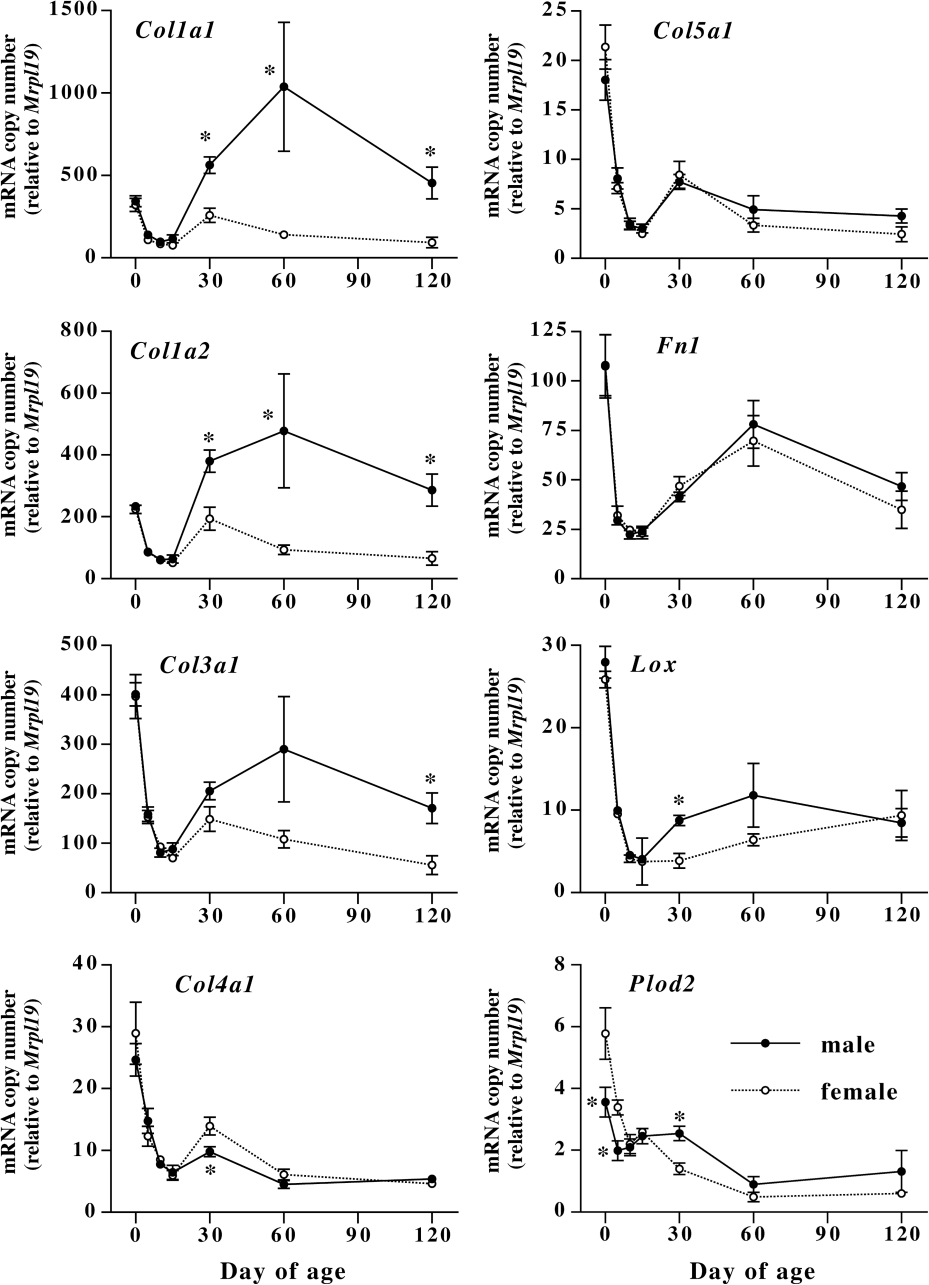
Postnatal changes in extracellular matrix and collagen cross-linking enzyme mRNA levels in male and female mouse dorsal skin. Male (closed circles) and female (open circles) mouse skin was collected at days 0, 5, 10, 15, 30, 60, and 120 of age, and mRNA levels were measured by quantitative realtime PCR. Values are means ± SEM for five or six animals. *, significant as compared to female animals at the same age (*P*<0.05).

Fig 2 shows changes in mRNAs encoding types I (*Col1a1*, *Col1a2*), III (*Col3a1*), IV (*Col4a1*) and V (*Col5a1*) collagens, fibronectin (*Fn1*), lysyl oxidase (*Lox*) and procollagen-lysine,2-oxoglutarate 5-dioxygenase 2 (*Plod2*) in mouse skin during postnatal development. Expression of *Mmp1a*, *Mmp1b* and *Mmp13* mRNAs was too low to obtain reliable data. Expression of all extracellular matrix mRNAs is relatively high at birth, and decreased to low levels until 10 or 15 days of age. After 30 days of age, apparent sexual dimorphism was observed in type I collagen mRNAs. *Col1a1* and *Col1a2* mRNAs in male skin significantly increased at day 30 as compared to female, and remained at high levels until day 120, while those in female mice remained at relatively low levels during adulthood. *Col3a1* mRNA in male skin also showed significantly high expression as compared to female at day 120. Expression of *Col5a1* and *Fn1* mRNAs in the skin did not show any sex differences. Similarly, levels of *Col4a1* mRNA did not show noticeable sexual dimorphism although expression in female skin at day 30 was significantly higher than that in male skin. Developmental changes in *Col4a1* and *Col5a1* mRNAs in the skin were very similar to each other. After the postnatal decrease, both *Col4a1* and *Col5a1* mRNAs slightly increased at day 30, decreased again at day 60 and remained at low levels until day 120. Developmental changes in skin *Fn1* mRNA levels were very similar to those in *Col3a1* in male skin. After the decrease during the infant stage, *Fn1* mRNA increased until day 60, and slightly decreased at day 120. Levels of *Lox* showed similar changes to *Col4a1* and *Col5a1*, but there was a significant sex difference at day 30 as observed in *Col1a1* and *Col1a2*. *Plod2* mRNA decreased after birth until day 60 in both male and female. Expression of *Plod2* mRNA was significantly low at days 0 and 5, and significantly high at day 30 in male as compared to female.

**Fig 3:**
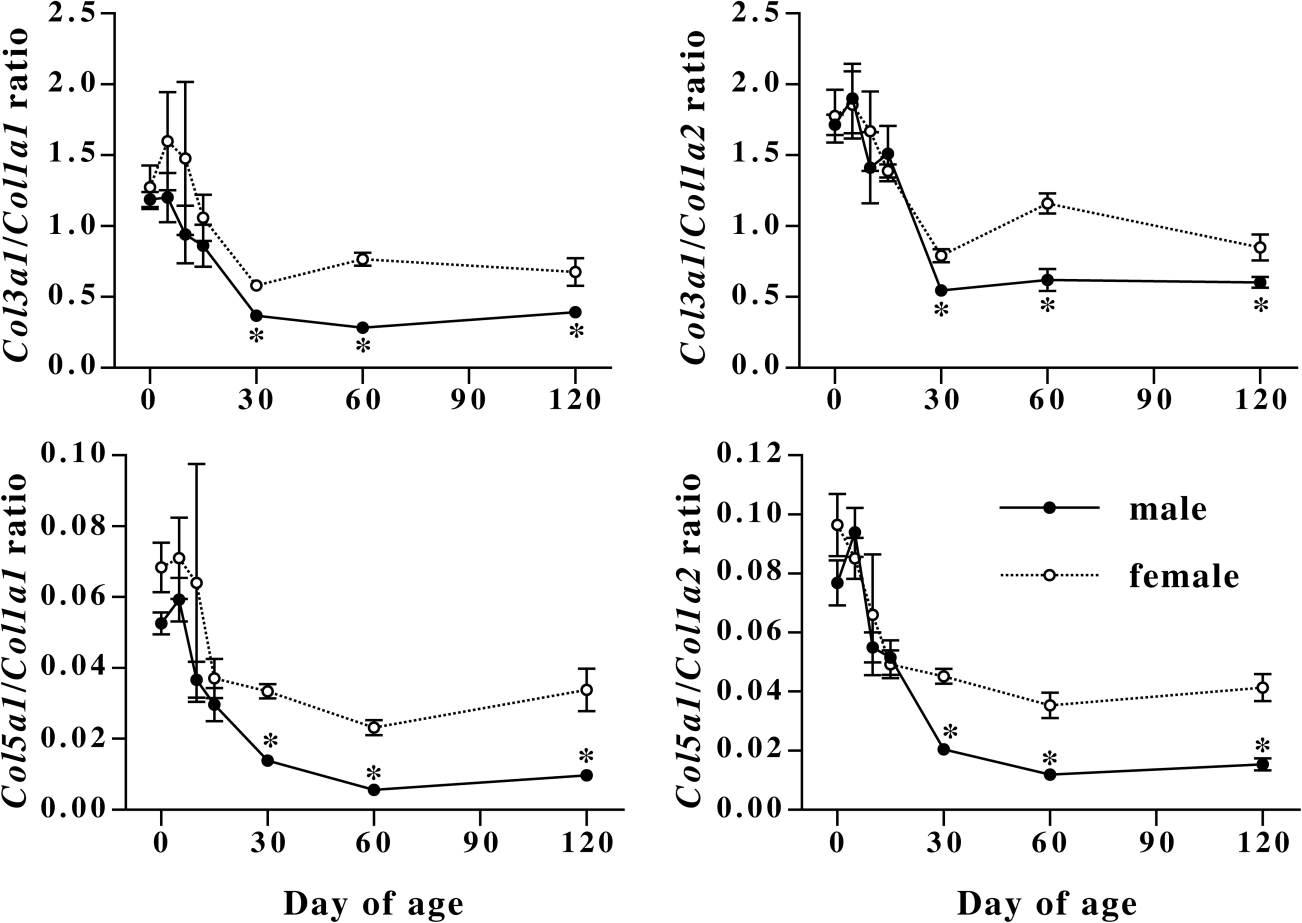
Changes in ratios of *Col3a1* and *Col5a1* mRNA levels to *Col1a1* and *Col1a2* mRNA levels in male and female mouse dorsal skin during development. Male (closed circles) and female (open circles) mouse skin was collected at days 0, 5, 10, 15, 30, 60, and 120 of age, and mRNA levels were measured by quantitative realtime PCR. Values are means ± SEM for five or six animals. *, significant as compared to female animals at the same age (*P*<0.05).

Because ratio of type III or type V collagen to type I collagen affects characteristics of collagen fibrils [9, 13, 25], ratios of *Col3a1* and *Col5a1* mRNAs to *Col1a1* and *Col1a2* mRNAs were calculated (Fig 3). During the first 15 days after birth, there was no sex difference in the ratios of *Col3a1* and *Col5a1* mRNAs to type I collagen mRNAs. However, after 30 days of age, the ratios in female mice showed significantly high values as compared to male.

### Postnatal changes in skin collagen contents in male and female mice

**Fig 4:**
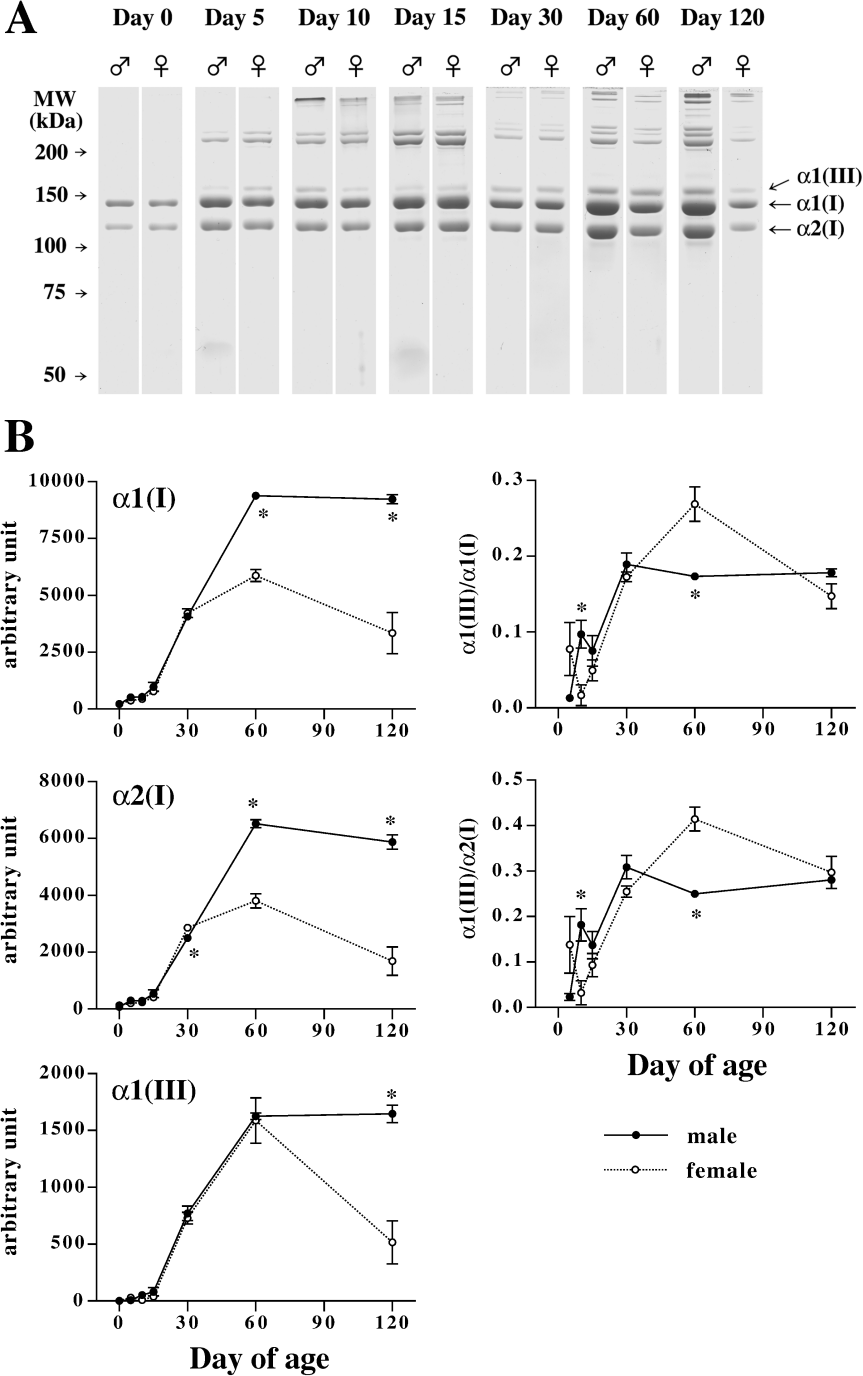
Changes in type I and type III collagen levels in mouse skin during development. (A) Representative images of SDS-PAGE of pepsin-treated male and female mouse dorsal skin samples collected at days 0, 5, 10, 15, 30, 60, and 120 of age. Samples were separated by 6% acrylamide gel containing 3.3 M urea under the reducing condition. The samples corresponding to 3 mg wet weight skin were loaded for days 0–15, and the samples corresponding to 0.35 mg wet weight skin were loaded for days 30–120. (B) Quantitative changes in collagen α1(I), α2(I), and α1(III) chains, and ratios of α1(III) chain to α1(I) and α2(I) chains in male (closed circles) and female (open circles) mouse dorsal skin during postnatal development. Intensity of bands in the SDS page was analyzed with Image J and normalized with the quantity of loaded samples. Values are means ± SEM for three animals. *, significant as compared to female animals at the same age (*P*<0.05).

Alpha1(I), α2(I) and α1(III) chains were separately detected by the SDS-PAGE in the presence of urea (Fig 4A). Changes in skin contents of type I collagen chains are consistent with the dermal thickness. As shown in Fig 4B, both α1(I) and α2(I) chains continuously increased during the first 30 days of age, and there is no sexual dimorphism during this period except for a significant but small difference in α2(I) chain at day 30. After 60 days of age, type I collagen contents considerably increased in male mouse skin as compared to female. With respect to type III collagen, α1(III) chain continuously increased during the first 60 days of age, and there is no sexual difference during this period. Skin contents of α1(III) chain remained at high levels in male mice while those in female mice significantly decreased as compared to male mice at day 120.

The bands for α1(III) chain were not detected at day 0. Therefore, ratio of α1(III) to α1(I) or α2(I) was calculated after 5 days of age (Fig 4B). Inconsistent with the ratios of mRNAs, the ratios of α1(III) chain to α1(I) and α2(I) chains were relatively low during infant stage, and increased from day 15 to day 30 in both male and female mouse skin. The ratios were significantly high in male as compared to female at day 10 of age. Thereafter, the ratios remained at constant values in male mice, while those in female mice temporally and significantly increased at 60 days of age as compared to male.

### Postnatal changes in expression of mRNAs encoding TGF-ßs, TGF receptors, activins, follistatin family proteins, and CTGF

**Fig 5:**
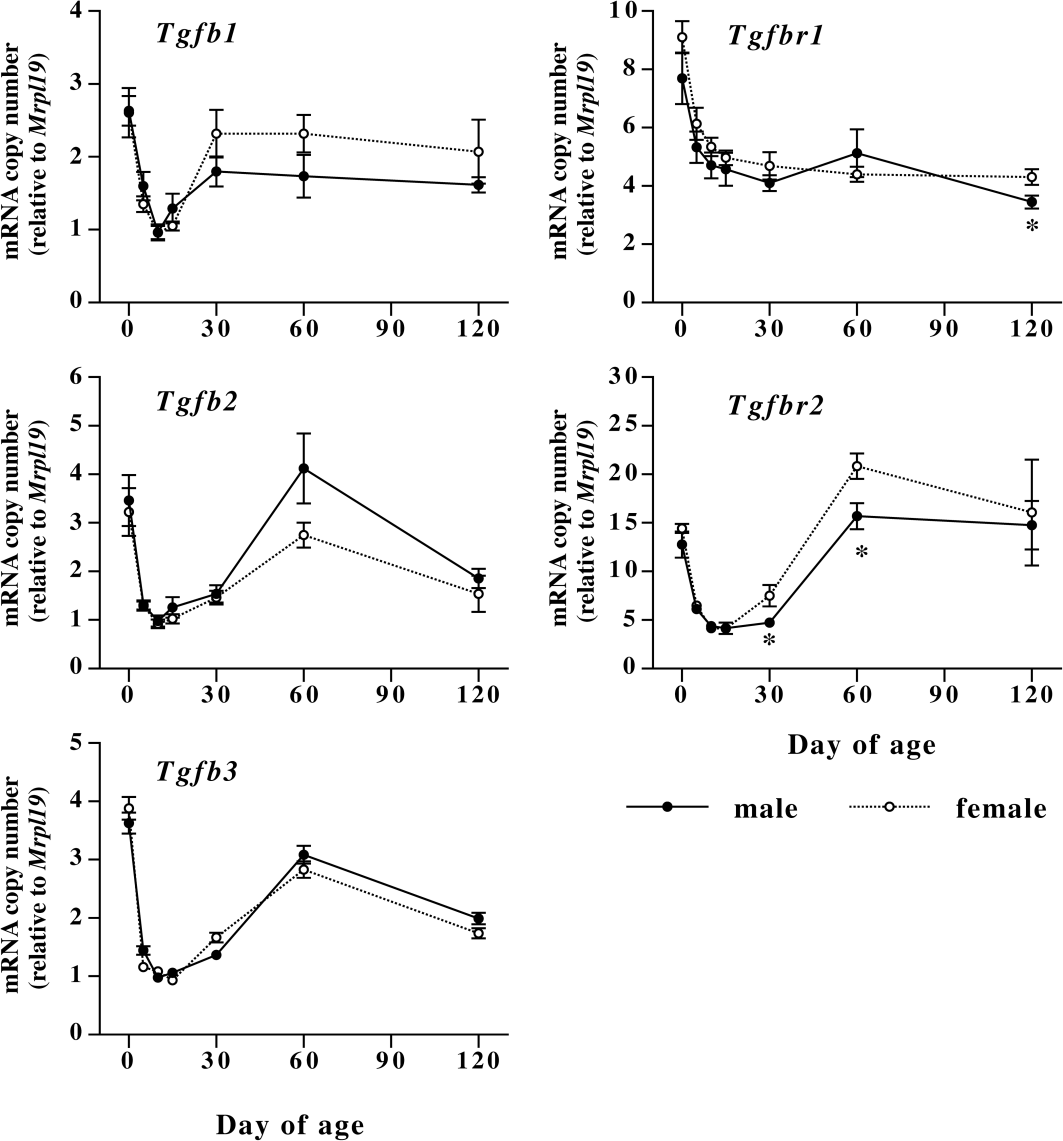
Postnatal changes in levels of mRNAs encoding TGF-ßs (*Tgfb1*, *Tgfb2* and *Tgfb3*) and their receptors (*Tgfbr1* and *Tgfbr2*) in male and female mouse dorsal skin. Male (closed circles) and female (open circles) mouse skin was collected at days 0, 5, 10, 15, 30, 60, and 120 of age. Values are means ± SEM for five or six animals. *, significant as compared to female animals at the same age (*P*<0.05).

**Fig 6:**
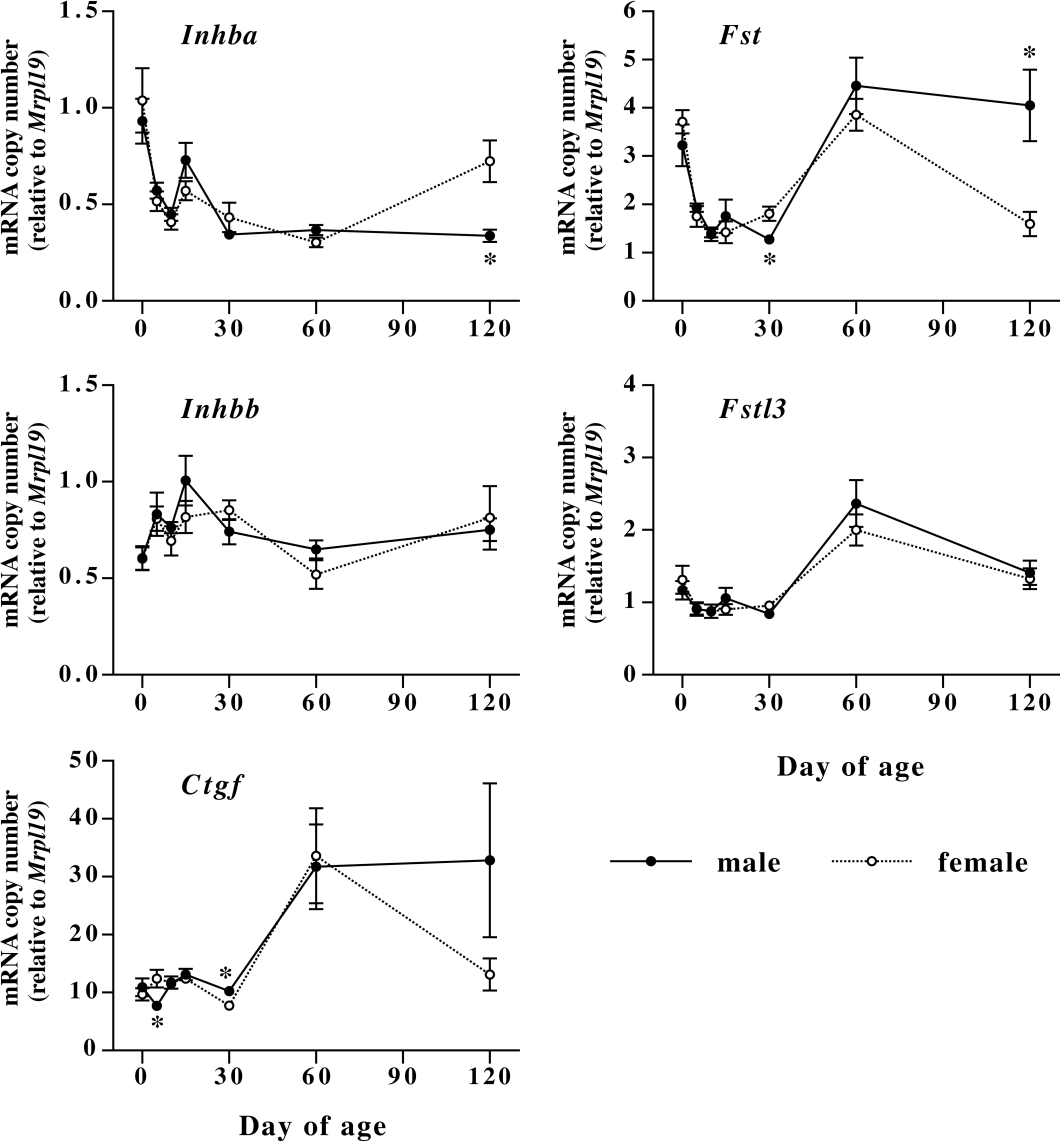
Postnatal changes in levels of mRNAs encoding activins (*Inhba* and *Inhbb*), follistatin family proteins (*Fst* and *Fstl3*), and CTGF (*Ctgf*) in male (closed circles) and female (open circles) mouse dorsal skin. Male (closed circles) and female (open circles) mouse skin was collected at days 0, 5, 10, 15, 30, 60, and 120 of age, and mRNA levels were measured by quantitative realtime PCR. Values are means ± SEM for five or six animals. *, significant as compared to female animals at the same age (*P*<0.05).

Postnatal changes in levels of mRNAs encoding TGF-ßs (*Tgfb1*, *Tgfb2*, *Tgfb3*) and their types I and II receptors (*Tgfbr1*, *Tgfbr2*) are shown in Fig 5, and the changes in mRNAs encoding activin subunits (*Inhba*, *Inhbb*), follistatin (*Fst*), follistatin-like 3 (*Fstl3*), and CTGF (*Ctgf*) are shown in Fig 6. As observed in extracellular matrix mRNAs, expression of mRNAs encoding TGF-ßs and their receptors is relatively high at birth, and decreased to low levels during infant stage. After the decrease, TGF-ß mRNAs and *Tgfr2* mRNA increased and remained at relatively high levels during adulthood. On the other hand, *Tgfbr1* mRNA remained at low levels after the postnatal decrease. There was no sex difference in expression of *Tgfb1*, *Tgfb2* and *Tgfb3* mRNAs in skin. Expression of *Tgfbr1* and *Tgfbr2* mRNAs did not show noticeable sex differences related to collagen expression although significant but small differences were observed at days 30, 60 and 120. Levels of *Inhba* mRNA were relatively high at birth, and rapidly decreased until day 10. In male mice, levels of *Inhba* mRNA remained at relatively low levels until day 120, while those in female mice significantly increased at 120 days of age as compared to male mice. *Inhbb* mRNA expression did not show either sexual dimorphism or apparent developmental changes. *Ctgf* mRNA levels remained at relatively low levels during the first 30 days after birth. There are significant differences in *Ctgf* mRNA levels between male and female during this period, but the differences are very small and did not correlate with collagen mRNA expression. After the period, expression of *Ctgf* mRNA increased at 60 days of age in both sexes. Thereafter, *Ctgf* mRNA returned to the infant levels in female and remained at high levels in male at 120 days of age, but the difference in *Ctgf* mRNA levels at day 120 was not significant. Expression of *Fst* mRNA was relatively high at birth, decreased to low levels until day 10, remained at the low levels until day 30, and increased to relatively high levels at 60 days of age. Thereafter, the mRNA expression remained at high levels in male, while those in female decreased to low levels. *Fst* mRNA expression in male skin was significantly lower than that in female at day 30 but the difference was very small. At day 120 of age, *Fst* mRNA expression in male skin was significantly higher than that in female. The levels of *Inhba* and *Fst* mRNAs at day 120 suggest that biological activity of activin A is high in female mice at this age as compared to male. With respect to *Fstl3* mRNA expression, there is no difference between male and female.

### Effects of castration on expression of skin fibril collagens, TGF-ßs, and TGF-ß receptors

**Fig 7:**
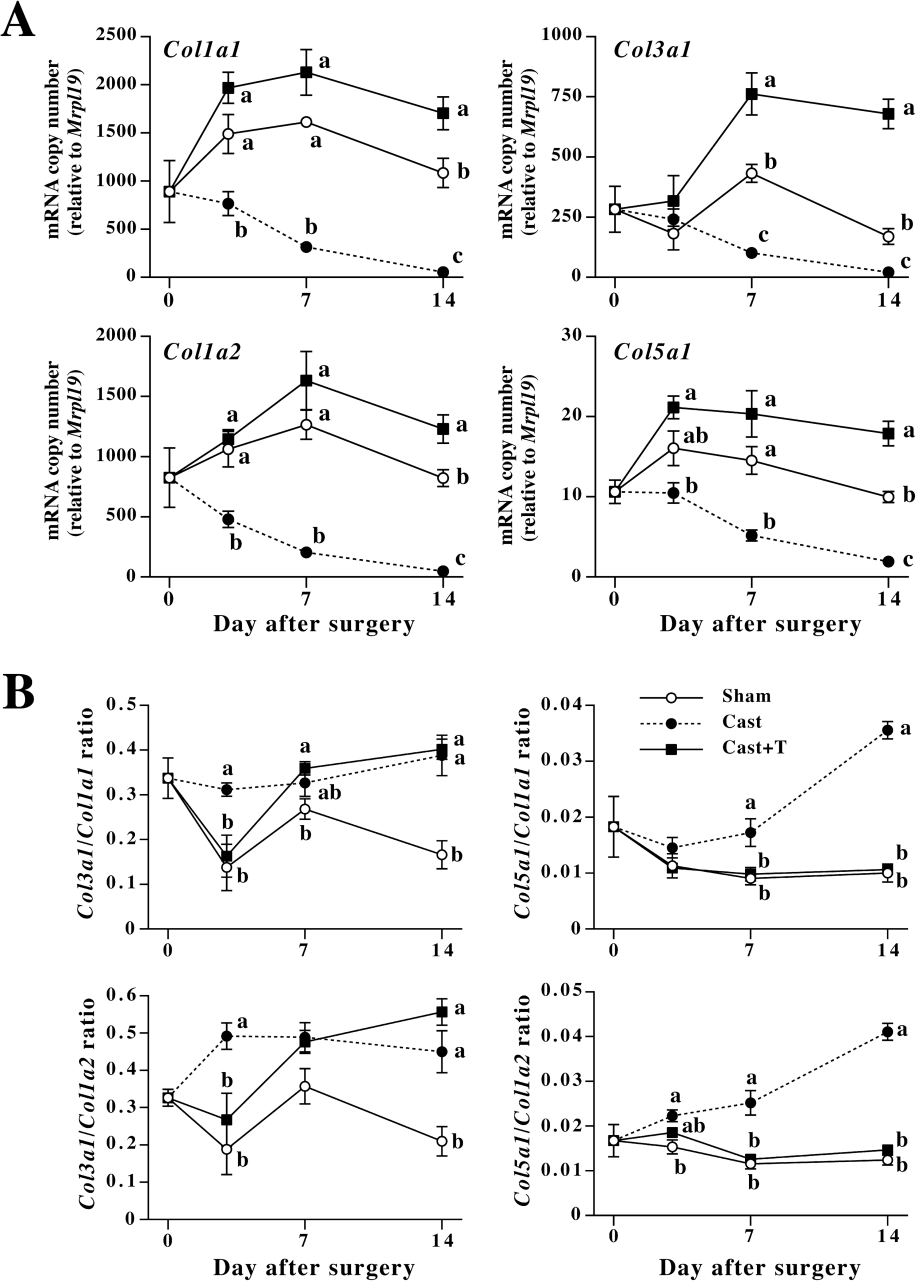
Changes in fibril collagen mRNA expression in mouse dorsal skin after castration. (A) Changes in levels of fibril collagen mRNAs after castration. (B) Changes in ratios of *Col3a1* and *Col5a1* to *Col1a1* and *Col1a2* after castration. Male mice were sham-operated or castrated at 60 days of age. Sham-operated (Sham, open circles) and a part of castrated mice (Cast, closed circles) were subcutaneously implanted with empty silicone tubes. A part of castrated mice were subcutaneously implanted with silicone tubes filled with testosterone crystal (Cast+T, closed squares). Dorsal skin of the mice was collected before and at 3, 7 and 14 days after the surgery, and mRNA levels were measured by quantitative realtime PCR. Values are means ± SEM for five or six animals. At each time point marked with letters, values without common letters are significantly different (*P*<0.05).

Castration at 60 days of age noticeably decreased weights of androgen-responsive organs seminal vesicles and coagulating glands (*P*<0.01), and implantation of the testosterone tube completely suppressed the decrease at 14 days after the surgery (sham-operated control group: 199.3 ± 17.2 mg, n = 6; castration group: 13.2 ± 1.9 mg, n = 6; castration+testosterone group: 325.4 ± 8.9, n = 6). The testosterone treatment significantly increased weights of the organs as compared to sham operation (*P*<0.01), indicating that the administration of testosterone achieved high concentration of circulating testosterone. As shown in Fig 7A, castration significantly decreased type I collagen mRNAs by 3 days after the surgery. Expression of *Col3a1* and *Col5a1* was also decreased by castration within 7 days. Administration of testosterone completely prevented the decrease in fibril collagen mRNA expression, and in some time points, significantly increased fibril collagen mRNAs as compared to sham-operated control animals. Implantation of dihydrotestosterone (DHT) tube showed the same effects on collagen expression as testosterone (data not shown), suggesting that the effects of testosterone are not due to its aromatized products. Consistent with the sexual dimorphism in ratios of *Col3a1* to type I collagen mRNAs and with ratios of *Col5a1* to type I collagen mRNAs, castration significantly increased the ratios as compared to control mice (Fig 7B). However, administration of testosterone only temporally suppressed the increase in ratios of *Col3a1* mRNA to type I collagen mRNAs, while testosterone treatment completely suppressed the increase in ratios of *Col5a1* mRNA to type I collagen mRNAs.

**Fig 8:**
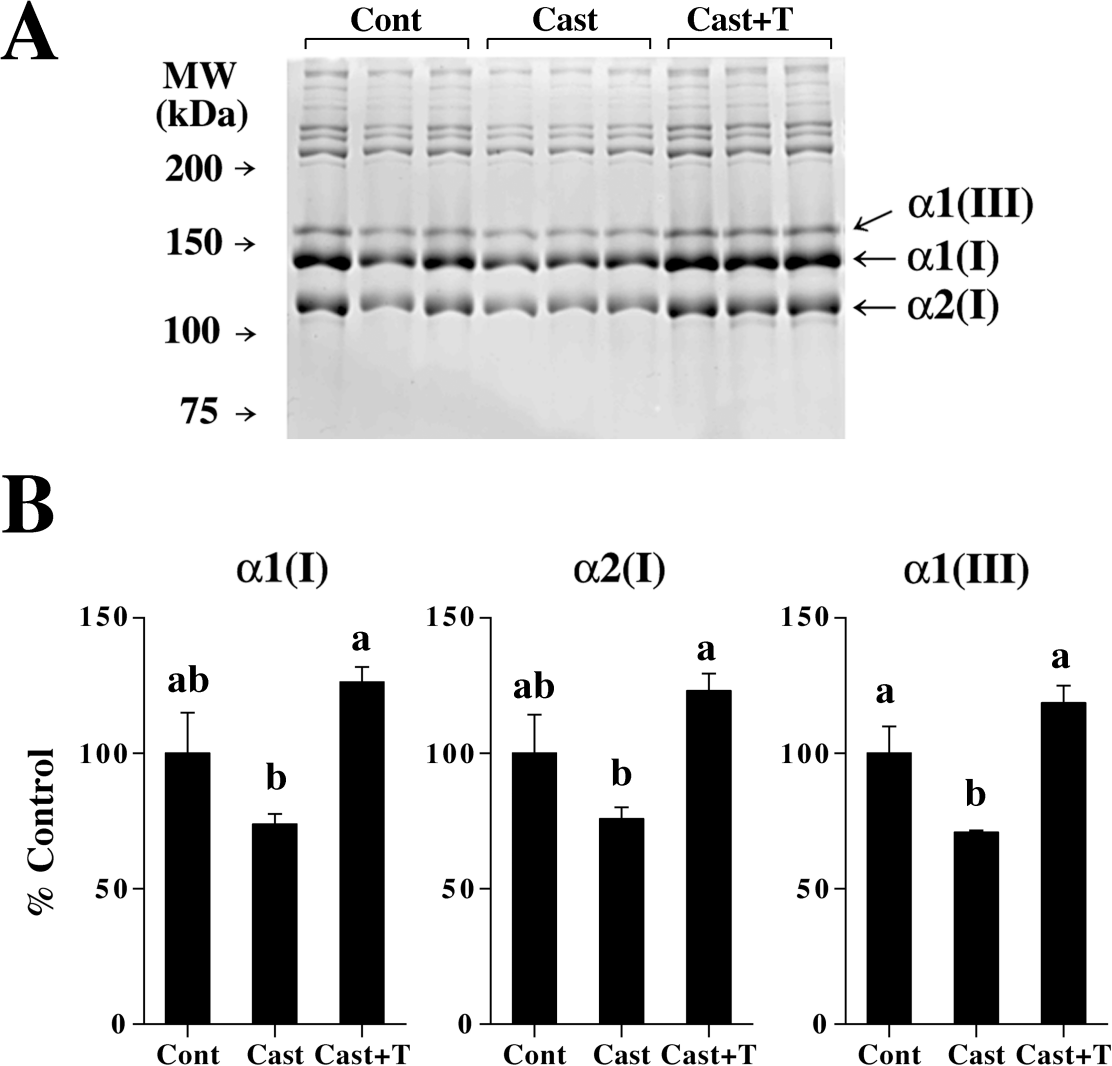
Types I and III collagen chains in castrated mouse skin. (A) An SDS-PAGE image of pepsin-soluble skin collagen chains in control mice (Cont), castrated mice (Cast), and castrated mice with testosterone treatment (Cast+T). Skin samples were collected at 14 days after the surgery. Samples were separated by 6% acrylamide gel containing 3.3 M urea under the reducing condition. The samples corresponding to 0.35 mg wet weight skin were loaded. (B) Relative levels of α1(I), α2(I), and α1(III) chains in the mouse skin. Intensity of bands was analyzed with Image J. Values are means ± SEM for three animals. Values without common letters are significantly different (*P*<0.05).

Castration tended to decrease α1(I) and α2(I) chains and significantly decreased α1(III) chain in skin at 14 days after the surgery (Fig 8). Administration of testosterone significantly increased types I and III collagen chains as compared to castrated mice without testosterone treatment.

**Fig 9:**
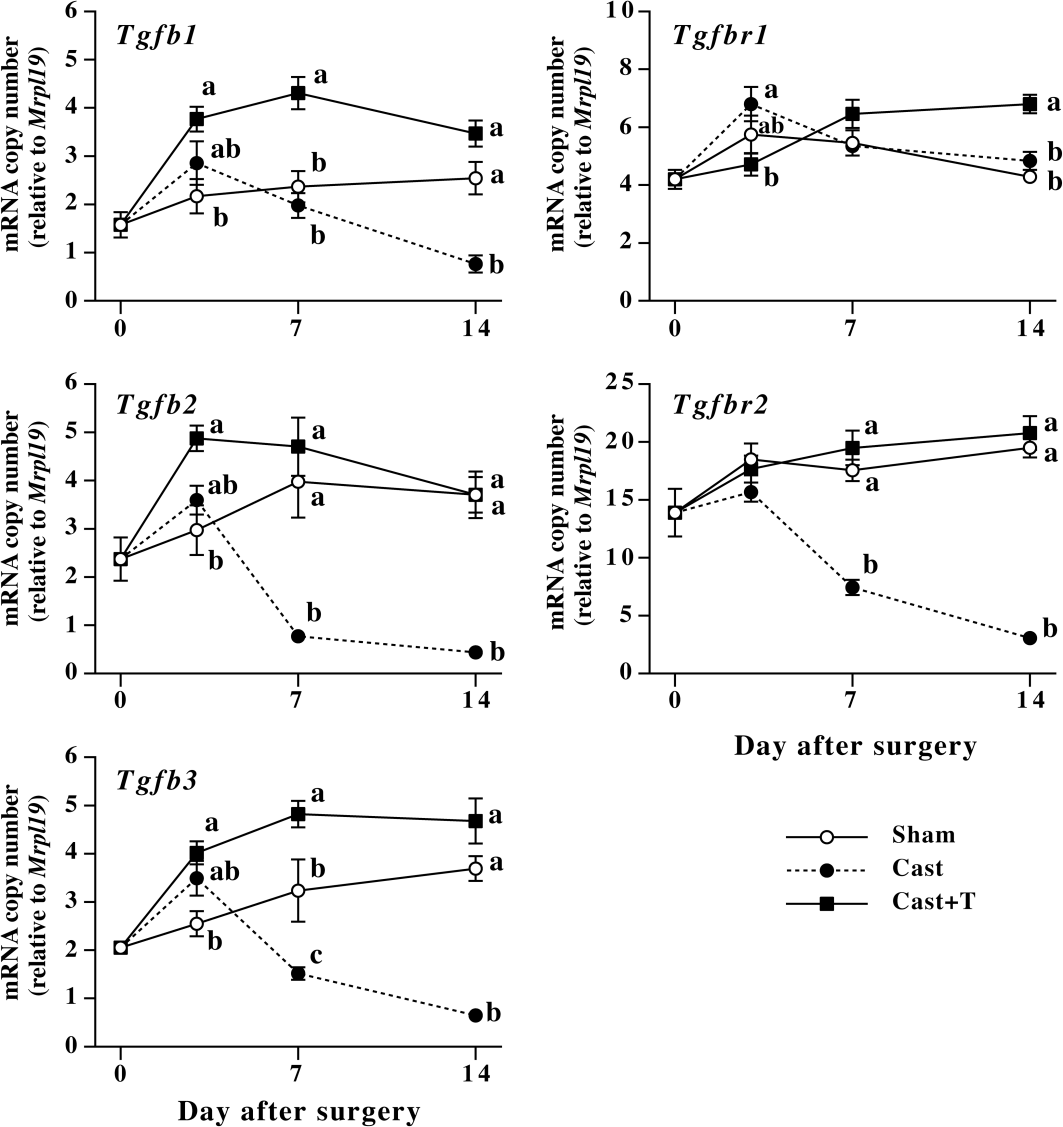
Changes in levels of mRNAs encoding TGF-ßs (*Tgfb1*, *Tgfb2* and *Tgfb3*) and TGF-ß receptors (*Tgfbr1* and *Tgfbr2*) in male mouse skin after castration. Male mice were sham-operated or castrated at 60 days of age. Sham-operated (Sham, open circles) and a part of castrated mice (Cast, closed circles) were subcutaneously implanted with empty silicone tubes. A part of castrated mice were subcutaneously implanted with silicone tubes filled with testosterone crystal (closed squares). Dorsal skin of the mice was collected before and at 3, 7 and 14 days after the surgery, and mRNA levels were measured by quantitative realtime PCR. Values are means ± SEM for five or six animals. At each time point marked with letters, values without common letters are significantly different (*P*<0.05).

Expression of mRNAs encoding TGF-ßs and type II TGF-ß receptor significantly decreased by castration by 7 or 14 days after the surgery (Fig 9). As observed in fibril collagen mRNAs, administration of testosterone completely prevented the castration-induced decrease in TGF-ß mRNAs and *Tgfbr2* mRNA, and at some time points, testosterone treatment significantly increased expression of these mRNAs as compared to control. With respect to *Tgfbr1* mRNA, castration did not affect its expression although administration of testosterone significantly increased the mRNA at 14 days after the surgery.

Despite the significant changes in pepsin-soluble collagen contents, either castration or androgen treatment did not affect dermal thickness at 14 days after the surgery (sham-operated control group: 310.1 ± 9.8 μm, n = 5; castration group: 343.7 ± 14.2 μm, n = 5; castration+testosterone group: 314.9 ± 13.9 μm, n = 5). Because castration significantly decreased dermal thickness at 1.5 months after the surgery [4], two weeks are probably not sufficient for detecting the effect of castration.

### Effects of testosterone on collagen expression in human and mouse fibroblasts

Human and mouse fibroblasts were treated with physiological concentrations of testosterone (1 and 5 ng/ml) for 24h, 72h or 1 week. However, testosterone did not affect fibril collagen mRNA expression in either human or mouse fibroblasts except at 1week treatment in human fibroblasts (S1 Fig). One-week treatment with 1ng/ml testosterone significantly increased *COL3A1* mRNA expression but the increase was not reproducible. These results suggest that the effects of testosterone on collagen expression *in vivo* are not due to a direct action of testosterone on dermal fibroblasts.

Function of fibroblasts is affected by keratinocyte-derived factors [26]. Therefore, we examined effects of testosterone on *COL1A1* mRNA expression in skin-equivalent, in which keratinocytes and fibroblasts interact with each other. However, testosterone did not affect *COL1A1* mRNA expression in the culture system (S2 Fig).

## Discussion

Sexual dimorphism and developmental changes in skin features are generally recognized. However, either differences in expression of mRNAs encoding collagens and their regulating factors between male and female or changing patterns of extracellular matrix expression during postnatal development have not fully been studied. Our present study revealed that expression of extracellular matrix mRNAs showed dynamic changes during postnatal development and apparent sexual dimorphism in mature mice. In addition, expression of TGF-ßs and their related genes showed noticeable changes after birth. These data suggest that the changes in expression of these mRNAs are involved in the developmental changes in skin features.

Skin contents of types I and III collagens and expression of their mRNAs in mature mice in our study are consistent with previous studies showing skin contents of hydroxyproline [4, 5] and type I collagen chains [4] in adult male and female mice, and demonstrated that the sexual dimorphism of collagen expression in mouse skin occurred at the mRNA level. The difference in fibril collagen mRNA expression between male and female mice started to be apparent at age of puberty. Circulating testosterone significantly increases around this age [27, 28], suggesting that testosterone is responsible for the sexual dimorphism in the fibril collagen expression. The castration experiments in this study strongly support the involvement of testosterone in the increase in skin collagen expression in male mice and demonstrated that the effect of testosterone appears within a few days. In addition, a previous study showed that ovariectomy did not affect expression of types I and III collagen mRNAs [4]. Estrogen stimulates collagen expression both in vivo and in vitro [29], but the sexual dimorphism in collagen expression suggests that testosterone is a more potent stimulator for collagen expression than estrogen under physiological conditions. The changes in skin contents of types I and III collagen chains indicate that a period of time is required for deposition of collagen fibrils to the dermis after the increase in gene expression. The present data also suggest that the different expression of types I and III collagens between male and female is a major factor responsible for the sex differences in skin features. The expression patterns of collagen cross-linking enzyme mRNAs suggest that cross-link modification of collagen fibrils may also be involved in the sex difference in skin contents of collagen. On the other hand, all extracellular matrix mRNAs examined in this study showed dynamic changes during development, suggesting that many extracellular matrices are involved in the developmental changes in skin features.

The changing patterns of TGF-ßs and their related genes during development suggest that the members of the TGF-ß superfamily are not likely involved in the sex difference in collagen expression in skin although castration largely reduced these mRNAs. Previous studies demonstrated that both androgen [30, 31] and estrogen [29, 32] stimulate expression of TGF-ßs. In female, estrogens may compensate for the stimulatory effects of testosterone on TGF-ßs during development. The castration-induced reduction of fibril collagen mRNAs occurred at earlier stage than that of TGF-beta mRNAs, suggesting that a decrease in TGF-ß signaling are not the main factor responsible for the castration-induced reduction of collagen mRNAs. On the other hand, the expression patterns of activin ßA subunit and follistatin suggest that activity of activin A in female mouse skin is higher than that in male mouse skin at day 120. Because activin stimulates extracellular matrix expression, the sex differences in *Inhba* and *Fst* expression are not likely responsible for the difference in collagen expression. Activin may be involved in other differences in skin features between mature male and female. With respect to the developmental changes, the relatively high expression of TGF-ß-related genes, as well as extracellular matrix genes, may be associated with skin constructing activity during neonatal skin development such as hair follicle growth, angiogenesis, and other accessory organ formation.

Type I collagen combines with type III and/or V collagens, and forms complex collagen fibril [8]. Previous studies demonstrated that the ratios of type III and/or V collagens to type I collagen affects collagen fibril diameter [9, 25]. In human skin, the proportion of type V collagen in total fibril collagen in adult skin is smaller than fetal skin [10]. Similarly, ratio of type III collagen to type I collagen decreases in human and rat skin as aging progressed [33, 34]. The changes in type III to type I or type V to type I ratio are thought to be responsible for the developmental changes in skin features at least in part. The present study indicates that changes in ratios of type III and type V collagen to type I collagen occur at the mRNA level. Furthermore, the present results demonstrated significant differences in the ratios of type III and type V collagen mRNAs to type I collagen mRNAs between male and female skin. These results suggest that the different proportions of collagen mRNA expression are partly responsible for sexual dimorphism in skin features. Changes in ratios of type III to type I collagen chains in the present study are not consistent with the previous observation in rat [12], in which the ratio decreased as aging progressed. In the present study, amount of pepsin soluble collagen was examined, while the previous study examined cyanogen bromide digested products. In addition, the SDS-PAGE method in this study does not analyze cross-linked collagen chains except for disulfide-linked collagen chains. The different results may be attributed to the difference in the analysis methods. The limitation of protein detection method in this study may explain the inconsistency between the type I/type III mRNA ratios and protein ratios. Castration also affected fibril collagen mRNA proportion. The results indicate that testosterone is an important factor regulating the ratio of type V to type I collagen. On the other hand, the ratios of *Col3a1* mRNA to type I collagen mRNAs did not depend on testosterone although castration significantly affected the ratio. The response of *Col3a1* mRNA to testosterone treatment was different from those of other fibril collagen mRNAs (Fig 7). The different response accounts for the changes in the ratios of *Col3a1* mRNA to type I collagen mRNAs in the castrated, testosterone-treated mice.

Despite the effects of testosterone on castrated mice, administration of testosterone did not affect fibril collagen expression in monolayer-cultured human and mouse dermal fibroblasts. It is contrastive to the effect of estradiol in vitro [29]. Although androgen receptor is localized in dermal fibroblasts [35], the present results indicate indirect action of testosterone on collagen expression *in vivo*. Epidermal keratinocyte was one of candidates for the target of testosterone involved in the effects of testosterone on collagen expression because keratinocytes strongly stained with anti-androgen receptor antibody [3] and because epidermal-dermal interaction has important roles in the regulation of skin homeostasis [26]. However, the results in the skin equivalent model denied the possibility. It is not clear whether the primary target of testosterone responsible for the increase in collagen expression exists in the skin. There are many humoral factors which directly stimulate collagen expression in fibroblasts. It remains to be determined whether the effects of testosterone on skin collagen expression are mediated by local factors in skin or by circulating hormone/growth factors.

In summary, the present study clearly demonstrated apparent sexual dimorphism in collagen mRNA expression and developmental changes in extracellular matrix and growth factor mRNA expression in mouse skin. Furthermore, stimulatory effects of testosterone on skin collagen mRNAs and TGF-ß mRNAs also demonstrated. However, the mechanism by which testosterone stimulates skin collagen expression remains to be determined.

## Supporting information

S1 Fig Effects of testosterone on fibril collagen mRNA expression in skin fibroblasts. Human (A) or mouse (B) skin fibroblasts were treated with testosterone at concentrations of 1 ng/ml (gray bars) and 5 ng/ml (solid bars) for 24h, 72 h, or 1 week. Values are representative of three independent experiments performed in triplicate. *, significant as compared to control (*P*<0.05).
(TIF)

S2 Fig Expression of *COL1A1* mRNA in skin equivalents in the presence or absence of testosterone. Skin equivalents were cultured for 14 days in the presence or absence of 5 ng/ml testosterone. After the incubation, dermal layers of the skin equivalents were collected and mRNA levels were examined by quantitative realtime PCR. Values are means ± SEM for three samples.
(TIF)

S1 Table List of primers used for realtime PCR. Mouse and human primers used for realtime PCR, related to Figs 2, 3, 5, 6, 7 and 9 and S1 and S2 Figs.
(DOC)
