# Supplementary figures and images for: Postnatal changes and sexual dimorphism in collagen expression in mouse skin
Source: PLoS One. 2017 May 11;12(5):e0177534. doi: 10.1371/journal.pone.0177534 (PMC5426772; doi:10.1371/journal.pone.0177534)

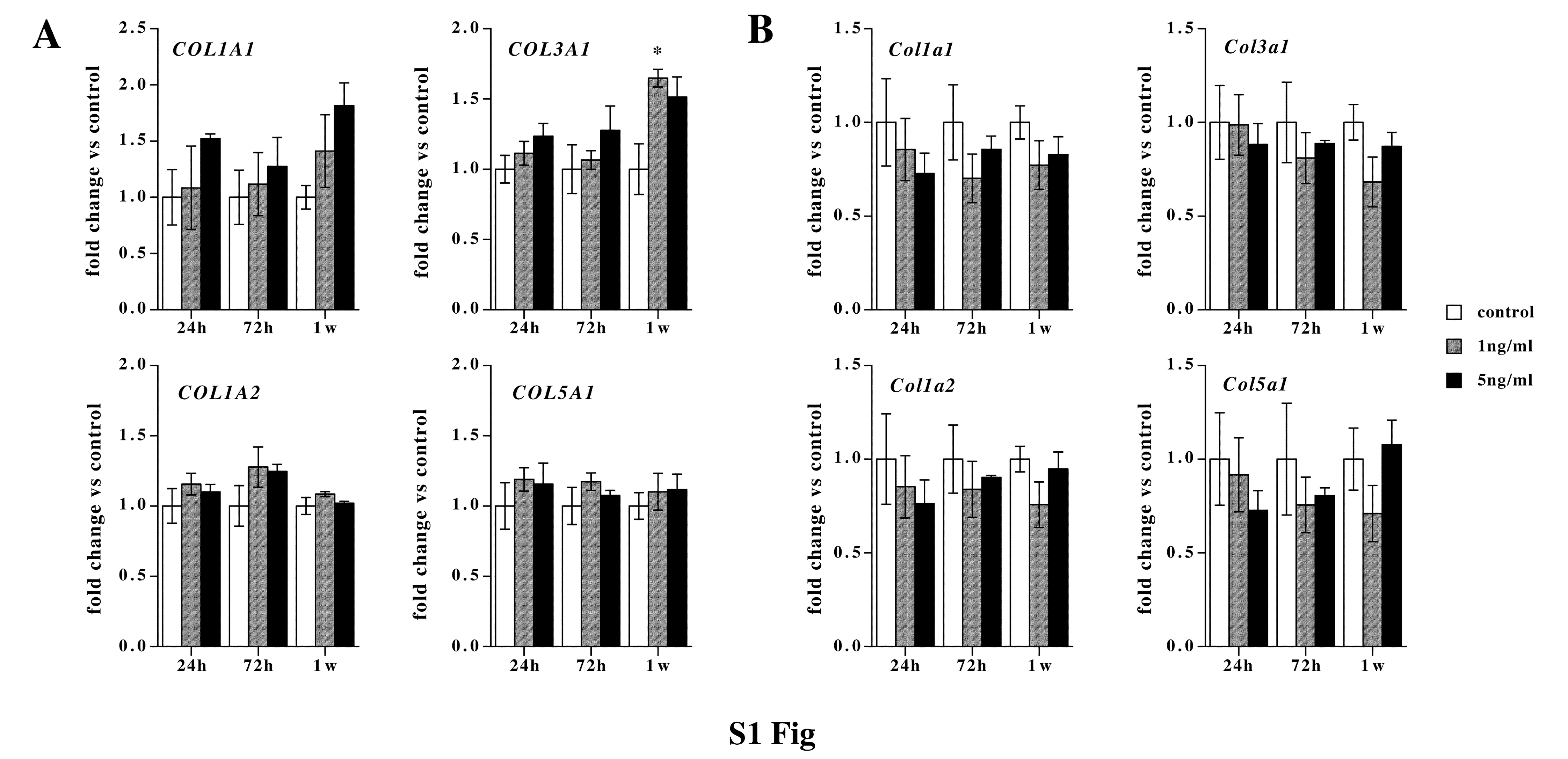

Supplement: S1 Fig — Human (A) or mouse (B) skin fibroblasts were treated with testosterone at concentrations of 1 ng/ml (gray bars) and 5 ng/ml (solid bars) for 24h, 72 h, or 1 week. Values are representative of three independent experiments performed in triplicate. *, significant as compared to control (P<0.05). (TIF) [file pone.0177534.s001.tif]

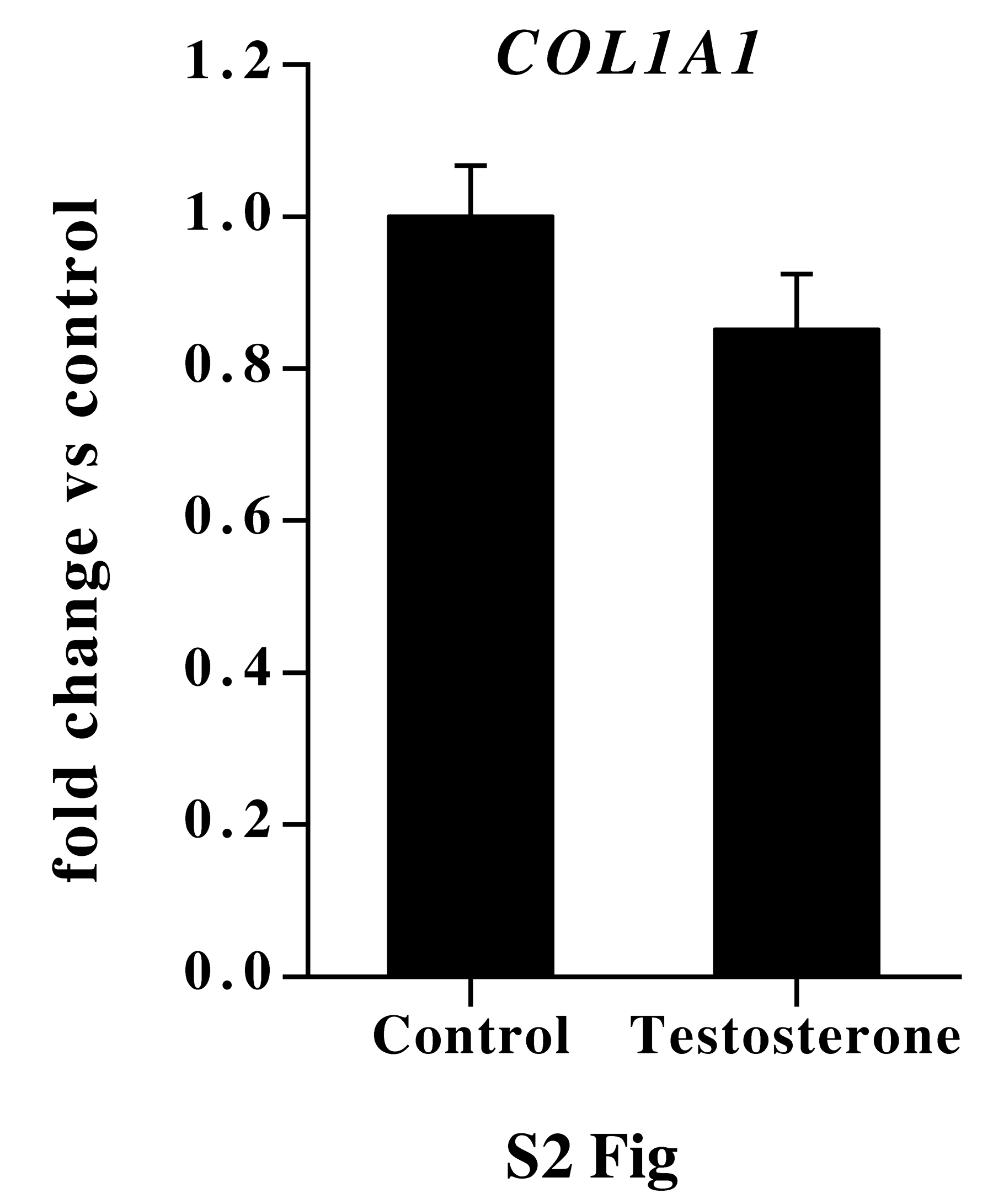

Supplement: S2 Fig — Skin equivalents were cultured for 14 days in the presence or absence of 5 ng/ml testosterone. After the incubation, dermal layers of the skin equivalents were collected and mRNA levels were examined by quantitative realtime PCR. Values are means ± SEM for three samples. (TIF) [file pone.0177534.s002.tif]
