# Supplementary material for: Postnatal changes and sexual dimorphism in collagen expression in mouse skin
Source: PLoS One. 2017 May 11;12(5):e0177534. doi: 10.1371/journal.pone.0177534 (PMC5426772; doi:10.1371/journal.pone.0177534)
Supplement: S1 Table — Mouse and human primers used for realtime PCR, related to Figs 2, 3, 5, 6, 7 and 9 and S1 and S2 Figs. (DOC) [file pone.0177534.s003.doc]

**S1 Table**. Primers used for the realtime PCR

|  |  | Upper primer | Lower primer |
| --- | --- | --- | --- |
| mouse | *Col1a1* | 5’-CCGAACCCCAAGGAAAAGA-3’ | 5’-CTGTTGCCTTCGCCTCTGA-3’ |
|  | *Col1a2* | 5’-CCGTTGGCAAAGATGGTAG-3’ | 5’-GTCAGCCCTGTAGAAGTATCC-3’ |
|  | *Col3a1* | 5’-AAAAACCCTGCTCGGAACT-3’ | 5’-CAGCTGCACATCAACGACA-3’ |
|  | *Col4a1* | 5’-AAGGGTGAGGCAGGAAAGG-3’ | 5’-CAGGCAAGCCATCAACACC-3’ |
|  | *Col5a1* | 5’-ATGGAGAAAGGGGTGACGA-3’ | 5’-GGCCATCCATACCCGTTAC-3’ |
|  | *Fn1* | 5’-CACCACCCAGAACTACGATG-3’ | 5’- GGGTCACACTTCCATCTGC-3’ |
|  | *Mmp1a* | 5’-CATCGCGAGAGCCTTTAGAGTC-3’ | 5’-GCCTGGCTGGAAAGTGTGA-3’ |
|  | *Mmp1b* | 5’-AAGGCGATATTGTGCTCTCC-3’ | 5’-CCTCATTGTTGTCGGTCCAC-3’ |
|  | *Mmp13* | 5’-TTGATGCCATTACCAGTCTCC-3’ | 5’-ATAAGGTCACGGGATGGATG-3’ |
|  | *Lox* | 5’-TGAAGAACCAAGGGACATCG-3’ | 5’-GCCTTCAGCCACTCTCCTCT-3’ |
|  | *Plod2* | 5’-ACCAACCCCCTTTCTACCTC-3’ | 5’-GTTCCTGGCTTCTGCTTGAC-3’ |
|  | *Tgfb1* | 5’-GCGGACTACTATGCTAAAGAGG-3’ | 5’-TCAAAAGACAGCCACTCAGG-3’ |
|  | *Tgfb2* | 5’-CTGTGCAGGAGTGGCTTCA-3’ | 5’-CGGCTGGACTGTTGTGACT-3’ |
|  | *Tgfb3* | 5’-CAAGAATCTGCCCACAAGG-3’ | 5’-TTGGGCTGAAAGGTGTGAC-3’ |
|  | *Tgfbr1* | 5’-GCAGCTGTGGTTGGTGTCA-3’ | 5’-TCTCACAGCAAGTCCCAAGTC-3’ |
|  | *Tgfbr2* | 5’-GCTCTGGTACTCTGGGAAATGACG-3’ | 5’-TGGATGCCCTGGTGGTTGAG-3’ |
|  | *Inhba* | 5’-GGAGAACGGGTATGTGGAGA-3’ | 5’-TGGTCCTGGTTCTGTTAGCC-3’ |
|  | *Inhbb* | 5’-CGAGATCATCAGCTTTGCAG -3’ | 5’- TCC ACC TTC TTC TCC ACC AC -3’ |
|  | *Fst* | 5’-TTCAAGTGGATGATTTTCAACGG-3’ | 5’-GTTGCGGTAGGTTTTCCCATC-3’ |
|  | *Fstl3* | 5’-TGCCCAGTACCTTCCAACC-3’ | 5’-TTCTCTTCCTCCTCTGCTGGTA-3’ |
|  | *Ctgf* | 5’-GGAAATGCTGCGAGGAGT-3’ | 5’-CTCGCATCATAGTTGGGTCTG-3’ |
|  | *Mrpl19* | 5’-CACCGCCGAAACCTGTCA-3’ | 5’-AGAACTCTGGAATCGGAAGCAC-3’ |
| human | *COL1A1* | 5’-ACAGCCGCTTCACCTACAG -3’ | 5’- GGATGGAGGGAGTTTACAGG-3’ |
|  | *COL3A1* | 5’- CCTGCTGGAAAAGATGGAG -3’ | 5’- CTTAGCACCAGGGGATCCA -3’ |
|  | *COL5A1* | 5’- CGGGAATGGCGAGAACTACG -3’ | 5’- ATTCTGGCCCCTTCGGACTT -3’ |
|  | *MRPL19* | 5’-GGGATTTGCATTCAGAGATCAG-3’ | 5’-GGAAGGGCATCTCGTAAG-3’ |
